# Risk prediction models for oral frailty in older adults: a scoping review

**DOI:** 10.3389/fmed.2026.1868632

**Published:** 2026-07-10

**Authors:** Yongjun Chen, Huixian Tang, Bo Li, Liping Yuan

**Affiliations:** 1 Graduate School, Wannan Medical College, Wuhu, Anhui, China; 2 Department of Nursing, First Affiliated Hospital, Wannan Medical College, Wuhu, Anhui, China

**Keywords:** older adults, oral frailty, risk assessment, risk prediction model, scoping review

## Abstract

**Background:**

Oral frailty reflects age-related decline in oral function and is associated with systemic functional decline and adverse outcomes in older adults. Accurate risk identification may support nursing assessment, risk stratification, and targeted intervention. However, the quality, validation status, and transportability of existing oral frailty prediction models remain unclear.

**Objective:**

This scoping review aimed to systematically map existing oral frailty risk prediction models for older adults, with particular attention to model development methods, outcome definitions, retained predictors, validation strategies, predictive performance, risk of bias, and applicability to clinical or community settings.

**Methods:**

PubMed, Web of Science Core Collection, Embase, CINAHL, ProQuest, the Cochrane Library, China National Knowledge Infrastructure, Wanfang, VIP, and SinoMed were searched from inception to 10 April 2026. Eligible studies developed, validated, or updated multivariable prediction models for oral frailty in older adults. Data extraction was guided by CHARMS, and risk of bias and applicability were assessed using PROBAST.

**Results:**

Seventeen studies were included. Most studies were cross-sectional and geographically concentrated in China, and oral frailty definitions and assessment approaches varied across studies. The reported proportion of oral frailty ranged from 25.50 to 92.50%. Across the included studies, logistic regression formed the basis of model development, while nomograms were generally used to display the final prediction tools. Frequently retained predictors included age, nutrition-related factors, swallowing difficulty or choking, frailty or physical frailty, and denture-related factors. Although 15 studies reported some form of internal validation, external validation was available in only two. The reported area under the receiver operating characteristic curve ranged from 0.725 to 0.985. Although most studies reported calibration assessment and several evaluated clinical utility, all studies were judged to have a high overall risk of bias.

**Conclusion:**

Existing models showed acceptable to excellent apparent discrimination, but clinical use remains constrained by heterogeneous outcome definitions, a predominance of cross-sectional designs, high risk of bias, limited external validation, and uncertain generalizability. Future studies should adopt prospective multicenter designs, standardize outcome definitions and reporting, define prediction horizons, and perform external validation before routine implementation.

**Systematic review registration:**

The systematic review has been registered on the Open Science Framework. The unique identifier is 10.17605/OSF.IO/K2GM4 (https://doi.org/10.17605/OSF.IO/K2GM4).

## 1 Introduction

Oral frailty (OF) refers to an age-related loss of oral functional reserve, reflected in declines across several domains of oral function, such as mastication, occlusal force, swallowing, oral moisture, and the ability to maintain oral hygiene (1). Because it sits at the interface between oral function and systemic health, OF has become increasingly relevant as populations age. Previous evidence has estimated its overall prevalence among older adults at 34%, with higher rates observed in more advanced age groups (2). OF is also associated with adverse outcomes beyond the oral cavity, including malnutrition, physical frailty, sarcopenia, falls, disability, cognitive decline, poorer quality of life, and mortality (3, 4). Identifying older adults at high risk of OF is therefore important for timely screening, risk stratification, and preventive intervention.

Clinical prediction models are increasingly used to support health assessment, disease prevention, and individualized care, and several models have now been developed to estimate the risk of oral frailty in older adults. In practice, these tools may help healthcare professionals recognize high-risk individuals earlier and inform targeted nursing management and individualized intervention (5). However, the available models vary markedly in the predictors they include, the methods used for model development, the way they are presented, the extent of validation, and their potential clinical use. It remains uncertain whether these models have sufficient predictive performance, methodological robustness, and generalizability for broader application.

Because the evidence varied substantially in outcome definitions, study populations, modeling strategies, and validation approaches, a scoping review was more appropriate than quantitative pooling of model performance. This approach allowed us to describe the available evidence and identify methodological gaps in oral frailty prediction research. Following the methodological approach outlined by Arksey and O’Malley (6), we examined oral frailty risk prediction models for older adults, focusing on how the models were developed, which predictors were retained, how they were validated, how well they performed, and their risk of bias. This review may help clarify the current evidence base and inform future improvement of prediction models for early screening and clinical risk assessment.

## 2 Methods

### 2.1 Protocol and reporting guideline

The review process was structured according to Arksey and O’Malley’s approach to scoping reviews, and reporting followed the PRISMA extension for Scoping Reviews (PRISMA-ScR). Because this review aimed to map the evidence rather than estimate pooled effects, the PCC framework was used to define the review scope. The review protocol is available through the Open Science Framework (https://doi.org/10.17605/OSF.IO/K2GM4).

### 2.2 Research questions

To define the scope of the review, we used the PCC framework: the population was older adults, the concept was risk prediction models for oral frailty, and the context covered community, hospital, rural, and disease-specific settings.

We addressed the following review questions:

(1) What oral frailty risk prediction models in older adults have been developed, and what modeling approaches have been used?
(2) What types of predictors have been selected and incorporated into these models?
(3) How well do the existing models perform, and have they undergone internal or external validation?
(4) What methodological limitations, risks of bias, and barriers to clinical application remain in the current evidence, and what implications do they have for future research?

### 2.3 Inclusion and exclusion criteria

We included studies that met the following criteria:

(1) Studies of adults aged 60 years or older in which oral frailty was defined and assessed according to the criteria specified in each original study;
(2) Studies that developed, validated, or updated oral frailty risk prediction models in older adults;
(3) Studies using observational designs, including cohort, cross-sectional, and case–control studies;
(4) Models including at least two predictors. If a study reported both model development and external validation of an existing model, only the development component was included.

We excluded studies that met any of the following criteria:

(1) Studies for which the full text was published in a language other than Chinese or English;
(2) Duplicate publications or studies with unavailable full texts;
(3) Studies that examined only associated or risk factors for OF without developing, validating, or updating a prediction model.

### 2.4 Literature search strategy

A systematic search was conducted in PubMed, Web of Science Core Collection, Embase, CINAHL, ProQuest, the Cochrane Library, China National Knowledge Infrastructure, Wanfang Database, VIP Database, and Chinese Biomedical Literature Database (SinoMed) from database inception to 10 April 2026. For each database, we tailored the search by combining relevant subject headings with free-text keywords and by accounting for database-specific indexing and search functions. The main search terms included “aged,” “elderly,” “older adults,” “oral frailty,” “oral frailty syndrome,” “oral frail*,” “oral function decline,” “oral hypofunction,” “risk prediction model,” “predictive model,” “nomogram,” and “risk assessment tool.” Corresponding Chinese terms were used when searching Chinese databases. To improve retrieval completeness, we examined the reference lists of included studies and relevant reviews for further eligible records. Given that oral frailty prediction research remains an emerging field and terminology is not yet fully standardized, we adopted a broad search strategy to maximize retrieval sensitivity. Nevertheless, eligibility was restricted to studies that developed, validated, or updated multivariable prediction models, and studies that only examined associated or risk factors without constructing a prediction model were excluded. The full search strategy for each database is reported in Supplementary Table S1.

### 2.5 Study screening and data charting

Duplicate records were identified and removed in NoteExpress software (Beijing Aegean Sea Lezhi Technology Co., Ltd., Beijing, China). Two trained reviewers then independently screened the remaining records against the prespecified eligibility criteria. Screening was carried out in two stages: titles and abstracts were reviewed first, and the full texts of potentially relevant articles were subsequently examined to confirm eligibility.

For data charting, we used a standardized form informed by the CHARMS checklist developed by Moons et al. (7). Two reviewers independently collected information from each eligible study, including bibliographic details, country or region, study design, target population, follow-up or observation period, oral frailty incidence or prevalence, sample size, modeling approach, reporting and handling of missing data, validation strategy, model performance, retained predictors, and model presentation. Information that was unclear or unavailable was recorded as not reported, and no additional data were requested from study authors. Any differences in judgment during study screening or data charting were discussed by the two reviewers; unresolved issues were referred to a third reviewer for a final decision.

### 2.6 Methodological quality assessment

Two trained reviewers independently assessed each included study for risk of bias and applicability with the Prediction Model Risk of Bias Assessment Tool (PROBAST) (8). Although formal risk-of-bias appraisal is not routinely required in all scoping reviews, PROBAST was applied because this review focused specifically on prediction model studies.

The PROBAST assessment covers four domains of prediction model studies: participants, predictors, outcomes, and analysis. For each domain, judgments were assigned as low, high, or unclear risk on the basis of the signaling questions and the information reported in each study. A low-risk judgment was made when the relevant criteria were adequately met; a high-risk judgment was assigned when important methodological concerns were present; and unclear risk was recorded when the available information was insufficient for a definite assessment. Disagreements between the two reviewers were first discussed, and unresolved cases were referred to a third reviewer.

### 2.7 Data synthesis

Evidence from the included studies was synthesized using a descriptive and narrative approach rather than quantitative pooling. Information on study population, sample size, study design, modeling method, predictor categories, model presentation, validation approach, and model performance was organized in tables and summarized narratively. To support comparisons across studies, reported performance measures were classified as discrimination, calibration, or clinical utility. Because study populations, outcome definitions, predictor selection, and modeling methods varied substantially, we did not perform a meta-analysis.

## 3 Results

### 3.1 Literature search and selection results

**Figure 1:**
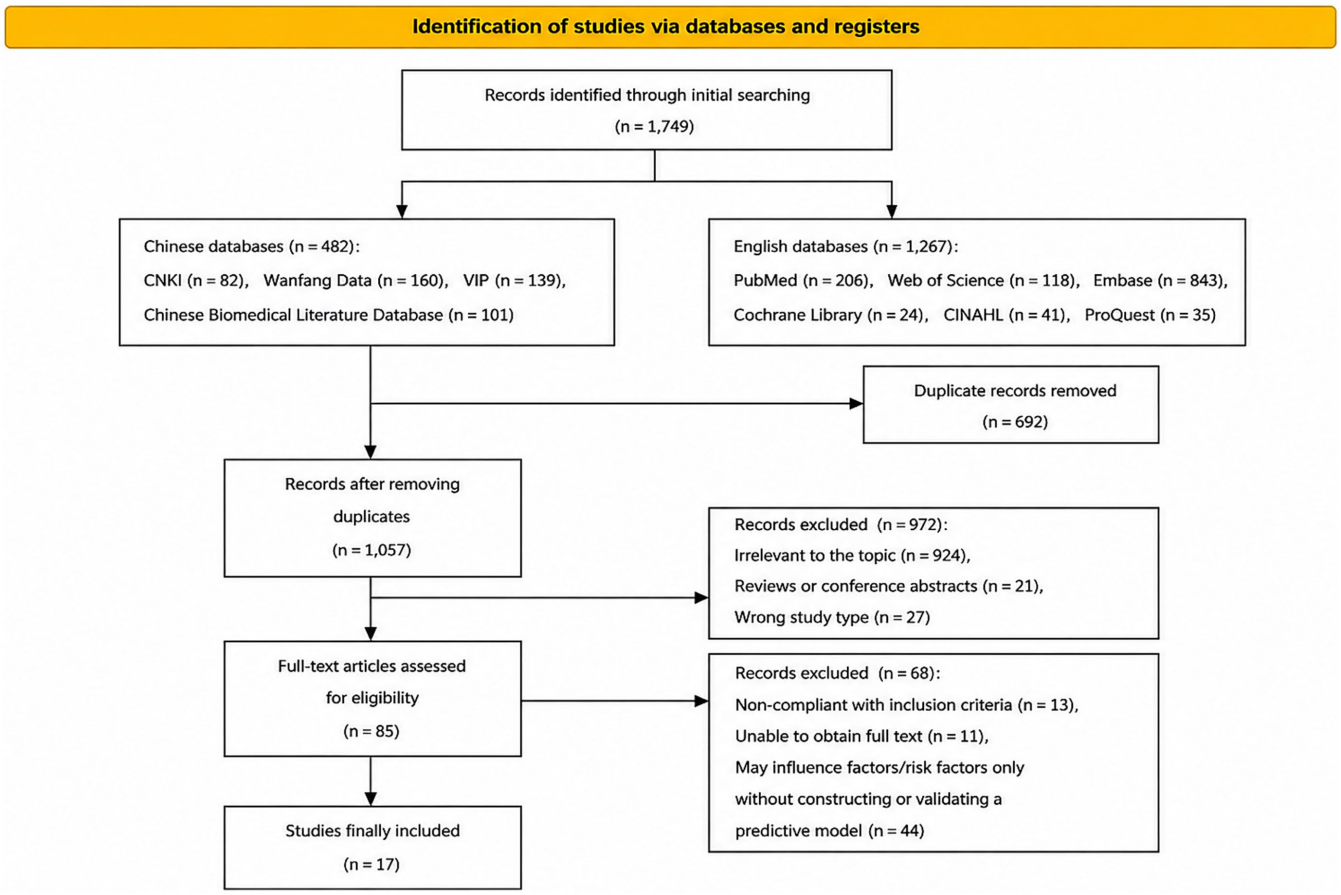
Preferred reporting items for systematic reviews (PRISMA) flowchart of literature search and selection.

The initial database search yielded 1,749 records. After 692 duplicates were removed, 1,057 unique records were screened by title and abstract. A total of 972 records were excluded during this stage, including records unrelated to the review topic (*n* = 924), reviews or conference abstracts (*n* = 21), and other records not taken forward to full-text assessment (*n* = 27). The full texts of 85 potentially relevant reports were then examined, and 68 were excluded after detailed assessment because they did not meet the inclusion criteria (*n* = 13), the full text was unavailable (*n* = 11), or no prediction model was developed or validated (*n* = 44). Finally, 17 studies were included in this review (9–25). The study selection process is summarized in Figure 1.

### 3.2 Characteristics of the included studies

The 17 included studies were published between 2022 and 2026; 16 were conducted in China and one in Japan. Cross-sectional designs accounted for most of the evidence. The study populations covered community-dwelling, rural, hospitalized, and disease-specific groups of older adults.

By population type, five studies enrolled community-dwelling older adults, one focused on rural older adults, and three included hospitalized older adults or hospitalized older adults with chronic diseases. The other eight studies examined older adults with specific diseases or clinical conditions, including stroke, Parkinson’s disease, chronic obstructive pulmonary disease, type 2 diabetes mellitus, cancer, esophageal cancer, and hypertension. Key characteristics of the included studies are summarized in Table 1 (9–25).

**Table 1:** Study characteristics and oral frailty occurrence across included studies.

Author	Year	Country	Design	Participants	Sample size	OF prevalence/incidence (%)
Jiang et al. (9)	2025	China	CS survey	Older adults	3,063; NG	46.82
Lu et al. (10)	2025	China	CS study	Older cancer patients	406; M 295/Val 111	M 64.41; Val 61.26
Guo and Xue (11)	2025	China	Retrospective	Older patients with Parkinson’s disease	214; M 150/Val 64	M 28.67
Li et al. (12)	2024	China	CS survey	Hospitalized older patients	780; M 546/Val 234	M 59.20; Val 59.40
Zou et al. (13)	2025	China	CS survey	Rural older adults	595; M 445/Val 150	M 50.3; Val NR
Wang et al. (14)	2025	China	CS survey	Community-dwelling older adults	548; Tr 383/Val 165	Overall 66.06; Tr 64.23; Val 70.30
Wu et al. (15)	2025	China	CS study	Community-dwelling older adults	586; M 411/Val 175	M 56.0; Val 50.3
Qiao et al. (16)	2025	China	CS study	Older patients with COPD	320; M 223/Val 97	92.5
Lv et al. (17)	2026	China	CS study	Older patients with esophageal cancer	555; Tr 390/Ext Val 165	Tr 45.90; Val 43.03
Feng et al. (18)	2026	China	CS study	Rural older patients with hypertension	538; M 377/Ext Val 161	61.5
Lv et al. (19)	2025	China	CS survey	Community-dwelling older adults	460; Tr 248/Val 106/Te 106	Overall 59.6
Huang et al. (20)	2026	China	CS study	Hospitalized older patients with chronic diseases	502; NG	68.92
Liu et al. (21)	2026	China	MC-CS study	Hospitalized older adults with chronic diseases	443; NG	69.3
Xiao et al. (22)	2026	China	CS study	Older patients with ischemic stroke	633; Tr 443/Val 190	Tr 63.2; Val 62.1
Yang et al. (23)	2026	China	CS study	Older adults with type 2 diabetes mellitus	533; Tr/Val = 7:3 random split	46.15
Ma et al. (24)	2025	China	CS study	Hospitalized older patients with stroke	664; M 451/Val 213	M 47.7; Val 47.9
Yamamoto et al. (25)	2022	Japan	CS study (SDA)	Older patients aged ≥65 years	843; Tr 595/Te 248	Overall 25.50; Tr 23.9; Te 29.4

OF, oral frailty; COPD, chronic obstructive pulmonary disease; CS, cross-sectional; MC-CS, multicenter cross-sectional; SDA, secondary data analysis; M, modeling set; Tr, training set; Val, validation set; Ext Val, external validation set; Te, test set; NG, not grouped; NR, not reported.

### 3.3 Risk of bias and applicability assessment

PROBAST was applied to all 17 studies to appraise risk of bias and applicability. All studies were rated as having high overall risk of bias. Applicability concerns were low in 16 studies (9–24) and high in one study (25). Study-level judgments are presented in Table 2, and the main sources of bias are discussed further in the Discussion section.

**Table 2:** PROBAST judgments for risk of bias and applicability.

Literature included	Participants ROB	Predictors ROB	Outcome ROB	Analysis ROB	Overall ROB	Overall applicability
Jiang et al. (9)	Low	Low	Low	High	High	Low concern
Lu et al. (10)	Low	Low	Low	High	High	Low concern
Guo and Xue (11)	Low	Low	Low	High	High	Low concern
Li et al. (12)	Low	Low	High	High	High	Low concern
Zou et al. (13)	Low	Low	High	High	High	Low concern
Wang et al. (14)	Low	Low	High	High	High	Low concern
Wu et al. (15)	Low	Low	High	High	High	Low concern
Qiao et al. (16)	Low	Low	Low	High	High	Low concern
Lv et al. (17)	Low	Low	Low	High	High	Low concern
Feng et al. (18)	Low	Low	High	High	High	Low concern
Lv et al. (19)	Low	Low	High	High	High	Low concern
Huang et al. (20)	Low	Low	High	High	High	Low concern
Liu et al. (21)	Low	Low	Low	High	High	Low concern
Xiao et al. (22)	Low	Low	High	High	High	Low concern
Yang et al. (23)	Low	Low	High	High	High	Low concern
Ma et al. (24)	Low	Low	Low	High	High	Low concern
Yamamoto et al. (25)	High	Low	High	High	High	High concern

ROB, risk of bias.

### 3.4 Overview of model development

Sample sizes across the included studies ranged from 214 to 3,063 participants, covering hospitalized, community-dwelling, and clinic-based older adults. Fourteen studies (10–19, 21–24) explicitly divided the datasets into development and validation cohorts, whereas three studies (9, 20, 25) did not clearly specify data partitioning. Logistic regression was the main modeling approach, including binary, multivariable, and stepwise multivariable logistic regression. Before final model building, some studies used variable-selection procedures such as correlation analysis (9), LASSO regression (11, 15, 17, 22), and random forest recursive feature elimination (22); nevertheless, most final prediction models were developed within a logistic regression framework. Detailed information on model development for each study is provided in Table 3.

**Table 3:** Model development methods, predictor selection, and presentation formats.

Included study	Model development method	Predictor selection method	No. of predictors	Final predictors included	Presentation format
Jiang et al. (9)	Binary logistic regression	Univariable analysis + correlation analysis + binary logistic regression	5	Age; disability; social isolation; sarcopenia; subjective cognitive decline	Nomogram
Lu et al. (10)	Logistic regression	Univariable analysis + logistic regression	7	Age; radiotherapy; oral mucositis; grip strength; poor oral health status; low oral health-related self-efficacy; high nutritional risk	Nomogram
Guo and Xue (11)	Multivariable logistic regression	LASSO regression + multivariable logistic regression	6	Age >70 years; primary or junior high school education; living alone; smoking; > = 2 chronic diseases; unbalanced diet	Nomogram
Li et al. (12)	Multivariable logistic regression	Univariable analysis + multivariable logistic regression	7	Age; dentures; > = 3 oral medications; dry mouth; frailty; nutrition; social support	Nomogram
Zou et al. (13)	Multivariable logistic regression	Univariable analysis + multivariable logistic regression	6	Age > = 80 years; polypharmacy; oral health-related self-efficacy; swallowing dysfunction; denture wearing; history of chronic diseases	Dynamic nomogram
Wang et al. (14)	Logistic regression	Literature review + univariable analysis + binary logistic regression	7	Age > = 80 years; removable denture wearing; reduced frequency of going out; toothbrushing <2 times/day; frequent dry mouth; greater difficulty eating hard foods; choking during eating	Nomogram
Wu et al. (15)	Logistic regression	LASSO regression + logistic regression	6	Passive smoking; living status; number of chronic diseases; physical frailty; dentures; nutritional status	Nomogram
Qiao et al. (16)	Multivariable logistic regression	Univariable analysis + multivariable logistic regression	3	Nutritional status; degree of dyspnea; type of chronic disease	Nomogram
Lv et al. (17)	Multivariable logistic regression	LASSO regression + multivariable analysis	6	History of radiotherapy; tumor stage; physical frailty; smoking history; age; nutritional status	Nomogram
Feng et al. (18)	Logistic regression	Univariable analysis + multivariable logistic regression	8	Age; education level; living alone; polypharmacy; smoking; dysphagia; dry mouth; comorbidity	Nomogram
Lv et al. (19)	Logistic regression	Univariable analysis + logistic regression	5	History of falls within 1 year; risk of sarcopenia; oral health knowledge; oral health beliefs; oral health behaviors	Nomogram
Huang et al. (20)	Multivariable logistic regression	Univariable analysis + multivariable logistic regression	7	Age; daily toothbrushing frequency; physical frailty; swallowing disorder; malnutrition; oral health status; social support	Nomogram
Liu et al. (21)	Binary logistic regression	Univariable analysis + binary logistic regression	5	Appetite; employment status; age; clinical physiological resilience; frailty	Nomogram
Xiao et al. (22)	Binary logistic regression	Univariable analysis + LASSO regression + RF-RFE + binary logistic regression	7	Denture wearing; toothbrushing frequency; xerostomia symptoms; chewing difficulty; swallowing function; oral health literacy; oral health status	Dynamic nomogram
Yang et al. (23)	Multivariable logistic regression	Univariable logistic regression + stepwise multivariable logistic regression	8	Age; type of chronic disease; duration of type 2 diabetes mellitus; HbA1c; periodontitis; number of natural teeth; difficulty chewing hard foods; swallowing disorder	Nomogram
Ma et al. (24)	Multivariable logistic regression	Univariable analysis + multivariable logistic regression	7	Age; frailty; number of comorbidities; nutritional risk; NIHSS; oral health assessment; Barthel index	Nomogram
Yamamoto et al. (25)	Stepwise multivariable logistic regression	Random 70/30 split + stepwise logistic regression	4	Age; number of remaining teeth; greater difficulty eating hard foods than 6 months earlier; recent choking when drinking tea or soup	Regression equation / questionnaire scoring model

LASSO, least absolute shrinkage and selection operator; RF-RFE, random forest recursive feature elimination; NIHSS, National Institutes of Health Stroke Scale; HbA1c, glycated hemoglobin A1c. Note: The number of predictors refers to the final predictors included in each prediction model.

### 3.5 Model presentation and predictors

Across the included studies, each final model incorporated three to eight predictors. Most models were presented as nomograms; two studies (13, 22) used dynamic nomograms, and one study (25) reported a regression equation/questionnaire-based scoring model. Predictors were grouped into four categories: general characteristics, laboratory indicators, disease-related factors, and other factors. The predictors retained most consistently across models were age, nutrition-related factors, swallowing dysfunction/choking, frailty or physical frailty, and denture use or removable dentures. Age appeared in 13 models, nutrition-related factors in 8, swallowing dysfunction/choking in 7, and frailty or physical frailty in 6. The distribution of predictor categories across models is summarized in Table 4.

**Table 4:** Predictor domains and their frequency across prediction models.

Category	Predictor	No.	Category	Predictor	No.
General characteristics	Age	13	Disease-related factors	Clinical physiological resilience	1
	Education level	2		Nutrition-related factors (nutritional status/risk, malnutrition, dietary balance)	8
	Living alone/residence status	3		Appetite	1
	Employment status	1		Comorbidity/chronic disease burden	4
	Disability	1		Type of chronic disease	3
	Reduced outings/activity	1		Duration of diabetes	1
	Smoking/passive smoking	4		Tumor stage	1
	History of falls	1		Periodontitis	1
Laboratory indicators	HbA1c	1		Oral mucositis	1
Disease-related factors	Dyspnea severity	1		Oral health status/poor oral health/assessment	4
	Stroke severity (NIHSS)	1	Other factors	Social support	2
	Activities of daily living (Barthel Index)	1		Social isolation	1
	Dentures/removable dentures	5		Subjective cognitive decline	1
	Number of teeth (remaining/natural teeth)	2		Oral health self-efficacy	2
	Toothbrushing frequency	3		Oral health knowledge	1
	Dry mouth/xerostomia	4		Oral health beliefs	1
	Difficulty chewing hard foods	4		Oral health behaviors	1
	Swallowing dysfunction/choking	7		Oral health literacy	1
	Frailty/physical frailty	6		History of radiotherapy	2
	Sarcopenia/sarcopenia risk	2			

### 3.6 Model validation and predictive performance

Among the 17 included studies, 15 reported some form of internal validation (10–19, 21–25), whereas external validation was conducted in only two studies (17, 18). Model discrimination was reported in all studies, and calibration was assessed in 16 studies (9–24). AUC estimates ranged from 0.725 to 0.985, indicating generally favorable discriminative ability. In the 16 studies that examined calibration, assessment was based mainly on the Hosmer–Lemeshow goodness-of-fit test, calibration curves, or both; most reported H–L test *p* values above 0.05 or close agreement between predicted and observed risks on calibration plots (9–24). Clinical utility was further examined using decision curve analysis in 12 studies (9, 11, 12, 14–18, 21–24). Study-level validation methods and predictive performance are summarized in Table 5.

**Table 5:** Validation strategies and predictive performance of oral frailty models.

Author	Validation method	Discrimination (AUC)	Calibration	Sensitivity	Specificity
Jiang et al. (9)	No grouping/no independent validation reported	0.725	H-L (*p* = 0.144); calibration curve; DCA (20–70%)	0.685	0.653
Lu et al. (10)	Validation set	Development/validation: 0.954/0.977	H-L (*χ^2^* = 0.439, *p* = 0.932)	Development/validation: 0.868/0.926	Development/validation: 0.895/0.907
Guo and Xue (11)	Validation set + bootstrap internal validation	Development/validation: 0.863/0.882	H-L (*χ^2^* = 5.282, *p* = 0.727); DCA (>8%)	Not reported	Not reported
Li et al. (12)	Validation set + bootstrap internal validation (1,000)	Development/validation: 0.866/0.887	Development: H-L (*χ^2^* = 9.069, *p* = 0.337); Validation: H-L (*χ^2^* = 7.678, *p* = 0.466); calibration curve; DCA	Development: 0.852; Validation: Not reported	Development: 0.715; Validation: Not reported
Zou et al. (13)	Validation set	Development/validation: 0.883/0.827	H-L (*χ^2^* = 6.31, *p* = 0.61); development/validation calibration curve	Development: 88.4%; Validation: 84.7%	Development: 72.9%; Validation: 75.6%
Wang et al. (14)	Enhanced bootstrap + validation set	Training: 0.950	H-L (*χ^2^* = 3.036, *p* = 0.932); calibration curve; DCA	0.91	0.85
Wu et al. (15)	Validation set + bootstrap internal validation (1,000)	Development/validation; internal validation: 0.952/0.936; 0.878	Development: H-L (*χ^2^* = 13.375, *p* = 0.100); Validation: H-L (*χ^2^* = 19.497, *p* = 0.112); calibration curve; DCA	Development: 89.6%; Validation: 86.4%	Development: 89.0%; Validation: 82.8%
Qiao et al. (16)	Validation set	Development/validation: 0.970/0.960	Development: H-L (*p* = 0.994); Validation: H-L (*p* = 0.540); calibration curve; DCA	Not reported	Not reported
Lv et al. (17)	Bootstrap internal validation (1,000) + external validation	Training/validation: 0.812/0.796	Training: H-L (*χ^2^* = 12.382, *p* = 0.193); Validation: H-L (*χ^2^* = 14.922, *p* = 0.093); calibration curve; DCA	Not reported	Not reported
Feng et al. (18)	Bootstrap internal validation (1,000) + external validation	Development; internal/external validation: 0.781; 0.769/0.810	H-L (*χ^2^* = 13.736, *p* = 0.089); calibration curve; DCA	0.819	0.648
Lv et al. (19)	Training/validation/test cohorts	Training/validation/test: 0.895/0.880/0.835	Training: H-L (*χ^2^* = 6.126, *p* = 0.633); Validation: H-L (*χ^2^* = 7.475, *p* = 0.486); Test: H-L (*χ^2^* = 5.603, *p* = 0.692); calibration curve	Not reported	Not reported
Huang et al. (20)	No grouping/no independent validation reported	0.846	H-L (*χ^2^* = 4.201, *p* = 0.839); calibration curve	0.694	0.853
Liu et al. (21)	Bootstrap internal validation (1,000; training/validation AUC also reported)	Training/validation: 0.805/0.900	H-L (*p* = 0.465, 0.161); calibration curve; DCA	Not reported	Not reported
Xiao et al. (22)	Bootstrap internal validation + validation set	Development/validation: 0.985/0.982	H-L; calibration curve; DCA	Development: 96.07%; Validation: 96.07%	Development: 93.87%; Validation: 93.75%
Yang et al. (23)	Bootstrap internal validation (1,000) + validation set	Development/validation: 0.847/0.831	H-L (χ^2^ = 13.548, *p* = 0.094); calibration curve; DCA	Development: 72.3%; Validation: 82.2%	Development: 83.5%; Validation: 72.4%
Ma et al. (24)	Validation set	Development/validation: 0.945/0.915	Development: H-L (χ^2^ = 5.632, *p* = 0.688); Validation: H-L (χ^2^ = 8.520, *p* = 0.384); DCA	Development: 88.2%; Validation: 88.2%	Development: 89.2%; Validation: 80.2%
Yamamoto et al. (25)	Random 70/30 training-test split	Test: 0.860	Not reported	Training: 0.94; Test: 0.90	Training: 0.67; Test: 0.66

AUC indicates the area under the receiver operating characteristic curve, H-L indicates Hosmer-Lemeshow, and DCA indicates decision curve analysis. Development, validation, training, internal validation, external validation, and test refer to the corresponding cohorts or validation datasets.

## 4 Discussion

### 4.1 Overall interpretation of the main findings

Our findings suggest that oral frailty risk prediction models for older adults have grown quickly in recent years and often report encouraging predictive performance. Compared with the previous systematic review (26), the available evidence now includes more studies and covers a broader range of populations and clinical settings. Even so, the apparent promise of these models needs to be weighed against several methodological concerns. Most studies (94.12%) were conducted in China, showing that the evidence remains geographically concentrated (9–24). In addition, only a small number of models had been examined in external datasets, and all included studies were rated as having high overall risk of bias. Overall, although existing models show potential, research in this area is still at an early stage.

### 4.2 Heterogeneity in oral frailty definitions and implications for model comparability

The heterogeneity of oral frailty definitions across the included studies deserves particular attention. Although all included studies focused on oral frailty or closely related constructs, the operational definitions, assessment tools, thresholds, and oral-function domains were not fully consistent (1, 2). Such variation may partly explain the wide range of reported oral frailty occurrence and may influence both predictor selection and apparent model performance. In particular, when retained predictors overlap with components used to define oral frailty, model performance may be overestimated and the transportability of the model to other populations may be limited (8). Therefore, comparisons across models should not rely solely on AUC values, but should also consider whether the outcome definition, target population, assessment method, and intended clinical context are comparable (8, 27).

### 4.3 Population heterogeneity and scenario-specific model development

The range of populations included in this review suggests that the risk structure of oral frailty is unlikely to be consistent across settings. As model development has moved beyond general older populations to community-dwelling, rural, hospitalized, and disease-specific groups, the intended use of these models has also become more varied. Models designed for community and rural older adults were generally oriented toward feasible early screening. Consistent with this aim, they more often included lifestyle, self-management, and oral health behavior-related factors, such as oral health-related self-efficacy, toothbrushing frequency, smoking or passive smoking, oral health knowledge, beliefs, and behaviors (13–15, 19). In contrast, models for hospitalized or disease-specific populations tended to incorporate condition-related, functional, or treatment-related indicators to support risk identification in more complex clinical contexts (10, 12, 16, 17, 20–24). For example, the model for older adults with COPD included dyspnea severity (16), whereas stroke-related models included swallowing function, oral health literacy, NIHSS score, and Barthel index (22, 24). Models for older adults with cancer or esophageal cancer also incorporated treatment or tumor-related factors, such as radiotherapy, oral mucositis, and tumor stage (10, 17).

This pattern suggests that oral frailty is unlikely to be a single fixed risk construct that can be fully captured by one universal model. Its risk profile appears to vary according to health background, care setting, and disease-specific characteristics. Future model development should therefore give greater weight to stratified and context-specific approaches tailored to different populations and application scenarios.

### 4.4 Interpretation of recurrent predictors and their clinical implications

Predictors that recurred across the included models suggest that oral frailty extends beyond local oral impairment. Age, nutrition-related factors, swallowing or choking-related variables, and frailty or physical frailty were repeatedly retained, suggesting that these domains may represent relatively stable core components of oral frailty risk. Age may capture cumulative declines in oral function, physiological reserve, and the ability to maintain daily oral self-care (28). The recurrent inclusion of nutrition and swallowing-related variables is also clinically relevant. Impaired chewing or swallowing may restrict food variety and reduce dietary intake, while prolonged nutritional insufficiency may further weaken muscle strength, physical function, and oral functional reserve (29). Similarly, the inclusion of frailty-related variables points to a close and potentially bidirectional relationship between oral frailty and general frailty. Declining oral function may worsen malnutrition and physical vulnerability, whereas physical frailty may limit an older adult’s ability to maintain oral hygiene, eating function, and self-care (30, 31).

Together, these findings support the interpretation of oral frailty within a broader geriatric framework rather than as an isolated oral condition. However, retained predictors should be interpreted with caution because systemic health status, nutritional condition, hospitalization, disease burden, and functional decline may act as predictors, confounders, mediators, or consequences of oral frailty (32–34). This issue is particularly relevant in hospitalized or disease-specific populations, where acute illness, treatment exposure, nutritional deterioration, and functional impairment may alter the risk structure of oral frailty (35). Therefore, future model development should combine recurrent core predictors with representative population-specific indicators, predefine candidate predictors based on clinical reasoning and theoretical plausibility, avoid inappropriate overlap between predictors and outcome components, and consider stratified or setting-specific development and validation to improve applicability across different settings.

### 4.5 Methodological limitations and risk of bias of existing models

Although all included models reported AUC values above 0.7, with estimates ranging from 0.725 to 0.985, this apparent performance should be viewed cautiously. Good discrimination or calibration in development datasets does not necessarily mean that a model is ready for routine clinical use. The clinical value of a prediction model depends not only on apparent performance, but also on sample representativeness, the strength of validation, and performance in independent populations (36). In this review, 88.24% of the studies did not include external validation, indicating that most oral frailty risk prediction models remain confined to model development or preliminary internal validation. This limitation is important because oral frailty may vary with regional background, disease profile, oral health behaviors, and access to healthcare resources (37, 38). Before wider implementation, these models should be externally validated in independent cohorts that represent diverse populations and care contexts.

Cross-sectional designs predominated among the included studies (9–25). Because predictors and oral frailty status were generally assessed at the same time point, the temporal interpretation of these models should be made with caution. In particular, it is difficult to determine whether candidate predictors preceded oral frailty or represented its consequences, correlates, or definitional components. Therefore, many existing models may be more appropriately interpreted as tools for current risk identification or case-finding rather than true prognostic models for incident oral frailty. This distinction has important implications for clinical implementation, as models derived from cross-sectional data may not be sufficient to predict future oral frailty or guide long-term preventive interventions. Before broader implementation, these models should be evaluated in prospective cohorts with clearly defined prediction horizons and externally validated in independent populations (39, 40).

The PROBAST assessment further showed that all included studies had high overall risk of bias, with concerns mainly related to the analysis domain and, in several studies, the outcome domain. Potential sources of bias included incomplete reporting or handling of missing data, non-standardized variable selection, inadequate control of overfitting, and limited external validation. Outcome-related bias may also have occurred in some studies because several final predictors overlapped with components used to define oral frailty, suggesting a possible risk of incorporation bias. Thus, the value of existing models should not be judged by AUC alone, but should be considered alongside study design quality, risk of bias, validation adequacy, and clinical utility.

### 4.6 Model development and methodological improvement

Most included models relied on logistic regression. This preference is understandable, as regression-based models are transparent, interpretable, and relatively easy to use in clinical screening (41). Oral frailty, however, is a multidimensional condition, and its risk may involve nonlinear relationships and interactions among demographic, functional, nutritional, disease-related, and behavioral factors (37). Conventional regression models may therefore have limitations when applied to complex or high-dimensional data.

Machine learning could add value in feature selection, pattern recognition, and model optimization (42). Recent studies have also explored interpretable methods, such as SHAP, in oral frailty risk prediction, pointing to a possible direction for future model development (43). This does not mean, however, that machine learning is inherently superior to traditional regression. Future studies should select modeling methods according to data characteristics, target populations, and intended clinical use. Predictive accuracy also needs to be considered alongside interpretability, feasibility, and clinical usability, so that models can be understood by healthcare professionals and incorporated into practical screening and intervention workflows.

### 4.7 Future research directions and implications for nursing practice

Future research should move beyond simply developing additional models and give greater emphasis to methodological robustness, validation quality, and practical clinical implementation. To strengthen model stability and generalizability, future studies should adopt prospective, multicenter designs and recruit sufficiently large samples. Candidate predictor selection, missing-data handling, model reporting, calibration assessment, and external validation also need to be more standardized. These steps would help ensure that prediction models are sufficiently reliable before being introduced into routine clinical practice (44).

In nursing practice, the value of prediction models lies not only in estimating risk, but also in supporting early identification, risk stratification, and targeted intervention (45). For older adults at high risk of oral frailty, possible interventions include oral health education, chewing and swallowing training, physical exercise, nutritional optimization, multidisciplinary collaboration, and family or social support (46). Nurses are well placed to contribute to screening, follow-up, health guidance, referral coordination, and intervention implementation. If future models are simplified and integrated into dynamic nomograms, web-based tools, electronic health systems, or routine nursing workflows, they may better support continuous assessment, risk stratification, and whole-process management of oral frailty in older adults.

### 4.8 Strengths and limitations

A notable strength of this review is that it specifically examined prediction models for oral frailty and used PROBAST to appraise their risk of bias. Several limitations should also be acknowledged. First, although we searched multiple Chinese and English databases and screened the reference lists of included studies and relevant reviews, we did not conduct a formal grey literature search, such as searches of dissertations, preprints, trial registries, institutional reports, or conference proceedings. Therefore, unpublished or non-indexed prediction model studies may have been missed, and publication bias cannot be fully excluded. Second, the included studies were largely concentrated in China, which may limit the generalizability of the findings to other countries and healthcare systems. Third, variation in oral frailty definitions and assessment methods across studies may have reduced the comparability and transportability of the models. Finally, incomplete reporting in some primary studies may have affected data charting and risk-of-bias judgments.

## 5 Conclusion

This review brings together the available evidence on prediction models for oral frailty in older adults. The findings indicate that model development in this field remains at an early and evolving stage. Although many models reported acceptable predictive performance, their clinical use is still limited by heterogeneous oral frailty definitions, a predominance of cross-sectional designs, high risk of bias, limited external validation, and insufficient evidence on stability, transportability, and real-world applicability. Many existing models may therefore be more suitable for current risk identification than for predicting incident oral frailty. Future studies should adopt more consistent and standardized approaches to outcome definition, model development, reporting, and validation, and should move from cross-sectional risk identification toward prospective prediction with clearly defined prediction horizons, multicenter external validation, and implementation testing across diverse care settings. Concise, interpretable, and generalizable prediction tools would better support early identification, nursing assessment, risk stratification, and targeted intervention for oral frailty in older adults.

## Data Availability

The original contributions presented in the study are included in the article/Supplementary material, further inquiries can be directed to the corresponding author.
