# Supplementary material for: Risk prediction models for oral frailty in older adults: a scoping review
Source: Front Med (Lausanne). 2026 Jul 10;13:1868632. doi: 10.3389/fmed.2026.1868632 (PMC13377978; doi:10.3389/fmed.2026.1868632)
Supplement: Supplementary file 1 [file Table_1.DOCX]

**Supplementary Table S1. Database search strategies**

*Risk prediction models for oral frailty in older adults: a scoping review*

**Search date:** 10 April 2026
**Search period:** From database inception to 10 April 2026
**Search concept blocks:** oral frailty/oral health; older adults; prediction/risk assessment/nomogram.

| **Database/source** | **Records** |
| --- | --- |
| **Chinese databases, total** | **482** |
| China National Knowledge Infrastructure (CNKI) | 82 |
| Wanfang Database | 160 |
| VIP Database | 139 |
| Chinese Biomedical Literature Database (SinoMed) | 101 |
| **English databases, total** | **1,267** |
| PubMed | 206 |
| Web of Science Core Collection | 118 |
| Embase | 843 |
| Cochrane Library | 24 |
| CINAHL | 41 |
| ProQuest | 35 |
| **Total records** | **1,749** |

# Database-specific search strategies

## 1. PubMed

(("oral frailty"[Title/Abstract] OR "oral weakness"[Title/Abstract] OR "oral frail"[Title/Abstract] OR "oral health"[Title/Abstract])
AND ("aged"[MeSH Terms] OR "older adults"[Title/Abstract] OR "senior citizen"[Title/Abstract] OR "advanced age"[Title/Abstract] OR "old people"[Title/Abstract])
AND ("risk prediction"[Title/Abstract] OR "risk assessment"[MeSH Terms] OR "prediction"[Title/Abstract] OR "nomograms"[MeSH Terms] OR "prediction rule*"[Title/Abstract] OR "prediction model*"[Title/Abstract] OR "prognosis model*"[Title/Abstract] OR "prediction*"[Title/Abstract]))

## 2. Web of Science Core Collection

TS=(("oral frailty" OR "oral weakness" OR "oral frail" OR "oral health")
AND (aged OR elderly OR "older adults" OR "senior citizen" OR "advanced age" OR "old people")
AND ("risk prediction" OR "risk assessment" OR prediction OR predict* OR nomogram* OR "prediction rule*" OR "prediction model*" OR "prognosis model*"))

## 3. Embase

('oral frailty':ti,ab,kw OR 'oral weakness':ti,ab,kw OR 'oral frail':ti,ab,kw OR 'oral health'/exp OR 'oral health':ti,ab,kw)
AND ('aged'/exp OR aged:ti,ab,kw OR elderly:ti,ab,kw OR 'older adult*':ti,ab,kw OR 'senior citizen*':ti,ab,kw OR 'advanced age':ti,ab,kw OR 'old people':ti,ab,kw)
AND ('risk prediction':ti,ab,kw OR 'risk assessment'/exp OR 'risk assessment':ti,ab,kw OR prediction:ti,ab,kw OR predict*:ti,ab,kw OR 'nomogram'/exp OR nomogram*:ti,ab,kw OR 'prediction rule*':ti,ab,kw OR 'prediction model*':ti,ab,kw OR 'prognosis model*':ti,ab,kw)

## 4. CINAHL

(MH "Oral Health+" OR TI "oral frailty" OR AB "oral frailty" OR TI "oral weakness" OR AB "oral weakness" OR TI "oral frail" OR AB "oral frail" OR TI "oral health" OR AB "oral health")
AND (MH "Aged+" OR TI aged OR AB aged OR TI elderly OR AB elderly OR TI "older adults" OR AB "older adults" OR TI "senior citizen" OR AB "senior citizen" OR TI "advanced age" OR AB "advanced age" OR TI "old people" OR AB "old people")
AND (MH "Risk Assessment+" OR MH "Nomograms" OR TI "risk prediction" OR AB "risk prediction" OR TI "risk assessment" OR AB "risk assessment" OR TI prediction OR AB prediction OR TI predict* OR AB predict* OR TI nomogram* OR AB nomogram* OR TI "prediction rule*" OR AB "prediction rule*" OR TI "prediction model*" OR AB "prediction model*" OR TI "prognosis model*" OR AB "prognosis model*")

## 5. ProQuest

NOFT(("oral frailty" OR "oral weakness" OR "oral frail" OR "oral health")
AND (aged OR elderly OR "older adults" OR "senior citizen" OR "advanced age" OR "old people")
AND ("risk prediction" OR "risk assessment" OR prediction OR predict* OR nomogram* OR "prediction rule*" OR "prediction model*" OR "prognosis model*"))

## 6. Cochrane Library

([mh "Oral Health"] OR "oral frailty":ti,ab,kw OR "oral weakness":ti,ab,kw OR "oral frail":ti,ab,kw OR "oral health":ti,ab,kw)
AND ([mh Aged] OR aged:ti,ab,kw OR elderly:ti,ab,kw OR "older adults":ti,ab,kw OR "senior citizen":ti,ab,kw OR "advanced age":ti,ab,kw OR "old people":ti,ab,kw)
AND ([mh "Risk Assessment"] OR [mh Nomograms] OR "risk prediction":ti,ab,kw OR "risk assessment":ti,ab,kw OR prediction:ti,ab,kw OR predict*:ti,ab,kw OR nomogram*:ti,ab,kw OR "prediction rule*":ti,ab,kw OR "prediction model*":ti,ab,kw OR "prognosis model*":ti,ab,kw)

## 7. China National Knowledge Infrastructure (CNKI)

主题 = ("口腔衰弱" OR "口腔虚弱" OR "口腔健康" OR "口腔功能下降" OR "口腔功能衰退" OR "口腔低功能" OR "口腔功能低下")
AND 主题 = ("老年" OR "老年人" OR "高龄" OR "老年患者" OR "老年群体")
AND 主题 = ("风险预测" OR "预测模型" OR "风险评估" OR "列线图" OR "诺模图" OR "预测" OR "风险评分" OR "评估工具")

**备注：**如 CNKI 专业检索界面不支持英文 OR，可将 OR 替换为“+”，并保留三组主题词之间的 AND 逻辑。

## 8. Wanfang Database

主题:("口腔衰弱" OR "口腔虚弱" OR "口腔健康" OR "口腔功能下降" OR "口腔功能衰退" OR "口腔低功能" OR "口腔功能低下")
AND 主题:("老年" OR "老年人" OR "高龄" OR "老年患者" OR "老年群体")
AND 主题:("风险预测" OR "预测模型" OR "风险评估" OR "列线图" OR "诺模图" OR "预测" OR "风险评分" OR "评估工具")

## 9. VIP Database

(题名或关键词=("口腔衰弱" OR "口腔虚弱" OR "口腔健康" OR "口腔功能下降" OR "口腔功能衰退" OR "口腔低功能" OR "口腔功能低下"))
AND (题名或关键词=("老年" OR "老年人" OR "高龄" OR "老年患者" OR "老年群体"))
AND (题名或关键词=("风险预测" OR "预测模型" OR "风险评估" OR "列线图" OR "诺模图" OR "预测" OR "风险评分" OR "评估工具"))

**备注：**维普高级检索界面可根据可用字段将“题名或关键词”替换为“任意字段/题名/关键词/摘要”的组合检索。

## 10. Chinese Biomedical Literature Database (SinoMed)

(("口腔衰弱"[常用字段] OR "口腔虚弱"[常用字段] OR "口腔健康"[常用字段] OR "口腔功能下降"[常用字段] OR "口腔功能衰退"[常用字段] OR "口腔低功能"[常用字段] OR "口腔功能低下"[常用字段])
AND ("老年"[常用字段] OR "老年人"[常用字段] OR "高龄"[常用字段] OR "老年患者"[常用字段] OR "老年群体"[常用字段])
AND ("风险预测"[常用字段] OR "预测模型"[常用字段] OR "风险评估"[常用字段] OR "列线图"[常用字段] OR "诺模图"[常用字段] OR "预测"[常用字段] OR "风险评分"[常用字段] OR "评估工具"[常用字段]))
